# Sex-disparities in chest pain workup: a retrospective cohort review of a university based clinical decision pathway

**DOI:** 10.1186/s12872-023-03610-3

**Published:** 2023-12-19

**Authors:** Benjamin R. Titus, Karen S. Ream, Tehreem Rehman, Larry A. Allen

**Affiliations:** 1 Internal Medicine Residency, University of Colorado, Aurora, United States; 2 Division of Cardiology, University of Colorado, Aurora, United States; 3 Department of Emergency Medicine, Mount Sinai Hospital, New York City, United States

**Keywords:** Acute chest pain, Sex-disparity, Clinical decision pathway, Non-invasive workup, HEART score

## Abstract

**Background:**

Females have historically lower rates of cardiovascular testing when compared to males. Clinical decision pathways (CDP) that utilize standardized risk-stratification methods may balance this disparity. We sought to determine whether clinical decision pathways could minimize sex-based differences in the non-invasive workup of chest pain in the emergency department (ED). Moreover, we evaluated whether the HEART score would minimize sex-based differences in risk-stratification.

**Methods:**

We conducted a retrospective cohort review of adult ED encounters for chest pain where CDP was employed. Primary outcome was any occurrence of non-invasive imaging (coronary CTA, stress imaging), invasive testing, intervention (PCI or CABG), or death. Secondary outcomes were 30-day major adverse cardiac events (MACE). We stratified HEART scores and primary/secondary outcomes by sex.

**Results:**

A total of 1078 charts met criteria for review. Mean age at presentation was 59 years. Females represented 47% of the population. Low, intermediate, and high-risk patients as determined by the HEART score were 17%, 65%, and 18% of the population, respectively, without any significant differences between males and females. Non-invasive testing was similar between males and females when stratified by risk. Males categorized as high risk underwent more coronary angiogram (33% vs. 16%, *p* = 0.01) and PCI (18% vs. 8%, *p* = 0.04) than high risk females, but this was not seen in patients categorized as low or intermediate risk. Males experienced more MACE than females (8% vs. 3%, *p* = 0.001).

**Conclusions:**

We identified no sex-based differences in risk-stratification or non-invasive testing when the CDP was used. High risk males, however, underwent more coronary angiogram and PCI than high risk females, and consequently males experienced more overall MACE than females. This disparity may be explained by sex-based differences in the pathophysiology driving each patient’s presentation.

**Supplementary Information:**

The online version contains supplementary material available at 10.1186/s12872-023-03610-3.

## Background

Chest pain is a common clinical presentation at Emergency Departments (ED) worldwide. Clinicians must quickly identify patients at high risk for acute coronary syndrome. Previously, they relied upon patient history and point-of-care troponin values to assess patient risk. This method historically led to lower rates of admission to the hospital and non-invasive testing in females, due in part to a combination of both biological and systemic factors [1]. Clinical decision pathways (CDPs) have emerged as a way to standardize the chest pain assessment and mitigate these disparities, but few studies have been published to evaluate this claim [2–5]. We set out to evaluate whether the CDP utilized at our academic institution minimized sex-based differences in risk-stratification, non-invasive versus invasive studies, interventions, and 30-day major adverse cardiac events (MACE).

## Methods

**Fig. 1:**
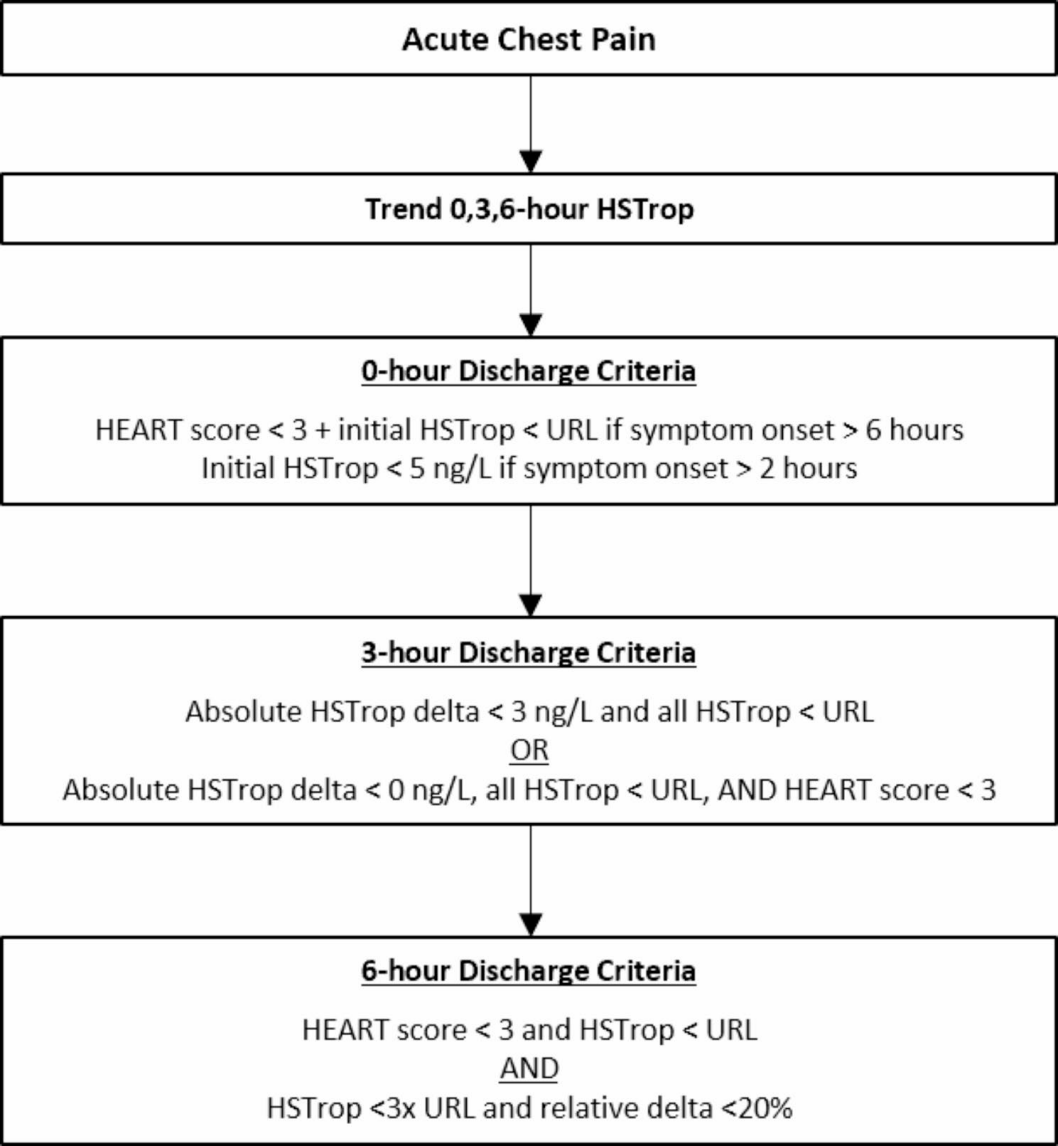
Clinical decision pathway discharge criteria. HSTrop = high-sensitivity troponin I or T, URL = 99th upper reference limit

We performed a retrospective analysis of patient encounters at our university-based ED that occurred between February 1, 2022 and July 31, 2022. We used database analytics to identify adult patients (≥ 18 years) with encounter diagnosis of chest pain (ICD-10 R07.X). In accordance with the institutional CDP, we included patients if at least two sequential high-sensitivity troponin values were checked during the encounter (Fig. 1). STEMI presentations were excluded from analysis as those presentations utilize a separate STEMI CDP. Additionally, patients were excluded if they left against medical advice prior to completion of workup or if ED practitioner documentation was incomplete.

Clinical variables necessary to calculate each patient’s HEART score were recorded [6]. Sex was defined as male or female. Chest pain descriptions as recorded by the ED providers were retrospectively categorized by a single reviewer as slightly suspicious, moderately suspicious, or highly suspicious. ECGs were retrospectively categorized as entirely normal, abnormal with repolarization abnormalities without significant ST depression/elevation, or abnormal ECG with significant ST deviation. Patient comorbidities were recorded, including hypertension, hyperlipidemia, diabetes mellitus, BMI > 30 kg/m^2^, smoking status, family history of premature CAD, personal history of CAD, prior MI, PCI/CABG, CVA/TIA, or PAD. High-sensitivity troponin scores were calculated by assigning 0 points for troponin less than the sex-specific upper limit of normal (URL), 1 point for troponin between the URL and URLx3, and 2 points for greater than the URLx3. High-sensitivity troponin analysis was performed on Access AccuTnI + 3 by Beckman Coulter with LOD < 2.3 ng/L and sex-specific upper limit of normal (URL) of 14.9 ng/L for females and 19.8 ng/L for males.

Primary outcome was any occurrence of coronary CTA, stress imaging such as stress echocardiogram or nuclear perfusion, coronary angiogram, percutaneous coronary intervention (PCI), CABG, or death for index encounter. Secondary outcome was any MACE (MI, stroke, or death) within 30-days. We stratified primary and secondary outcomes by HEART score risk category and sex. We then compared these outcomes using Chi-Squared test or Fisher’s Exact test as appropriate with α = 0.05. All analyses were performed with XLSTAT (Addinsoft, New York, USA).

## Results

**Fig. 2:**
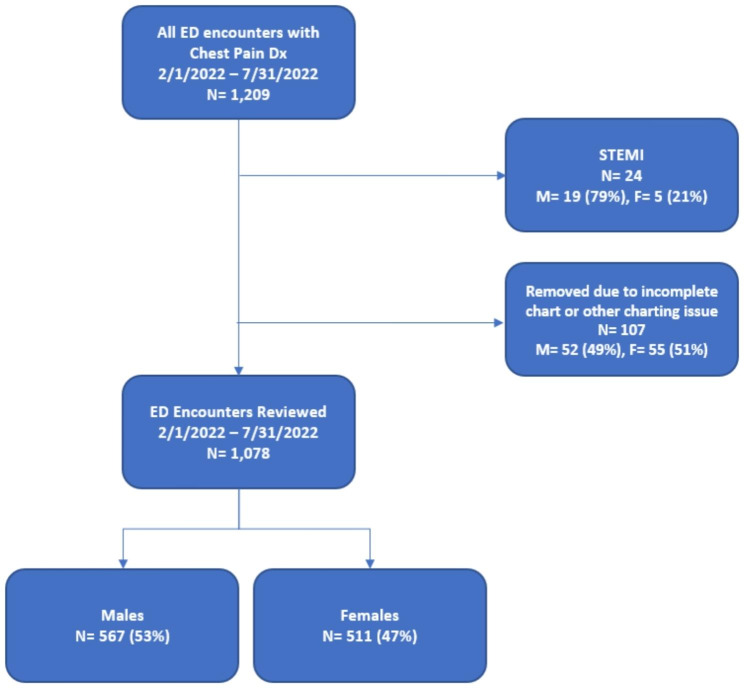
Cohort diagram illustrating chart inclusion/exclusion

A total of 1078 charts met criteria for review after exclusionary criteria were applied (Fig. 2). Females represented 47% of the population (Table 1). Mean age was similar between males and females at 59 years. More males identified as active smokers or having quit smoking than females. More males had a history of coronary artery disease, past MI, and family history of premature CAD than females. There were no other significant differences between baseline demographics for males and females. Low, intermediate, and high-risk patients as determined by the HEART score were 17%, 65%, and 18% of the population, respectively, without any significant differences between males and females (*p* = 0.52).

**Table 1:** Patient Cohort Characteristics

	Males (n = 567)		Females (n = 511)		*p*-value
**Heart Score, No. (%)**					
Low (0–3)	101	(18%)	85	(17%)	0.52
Intermediate (4–6)	362	(64%)	340	(67%)	
High (7+)	104	(18%)	86	(17%)	
**Age, years**	59.3		59.2		0.92
**BMI > 30, No. (%), kg/m** ^**2**^	249	(44%)	246	(48%)	0.16
**Smoking, No. (%)**					
Never/Passive	204	(36%)	262	(51%)	<0.0001
Quit	172	(30%)	134	(26%)	
Active	129	(23%)	80	(16%)	
Not asked/Missing	62	(11%)	35	(7%)	
**Comorbidities, No. (%)**					
CAD	94	(17%)	60	(12%)	0.02
Diabetes	145	(26%)	158	(31%)	0.051
Hypertension	317	(56%)	296	(58%)	0.50
Hyperlipidemia	147	(26%)	121	(24%)	0.39
History of TIA/Stroke	53	(9%)	62	(12%)	0.14
History of PCI/CABG	66	(12%)	45	(9%)	0.13
History of PAD	13	(2%)	7	(1%)	0.26
History of MI	99	(17%)	67	(13%)	0.048
Family history of CAD	46	(8%)	66	(13%)	0.01

BMI = body mass index; CAD = coronary artery disease; TIA = transient ischemic attack;

PCI = percutaneous coronary intervention; CABG = coronary artery bypass grafting;

PAD = peripheral arterial disease; MI = myocardial infarction

**Fig. 3:**
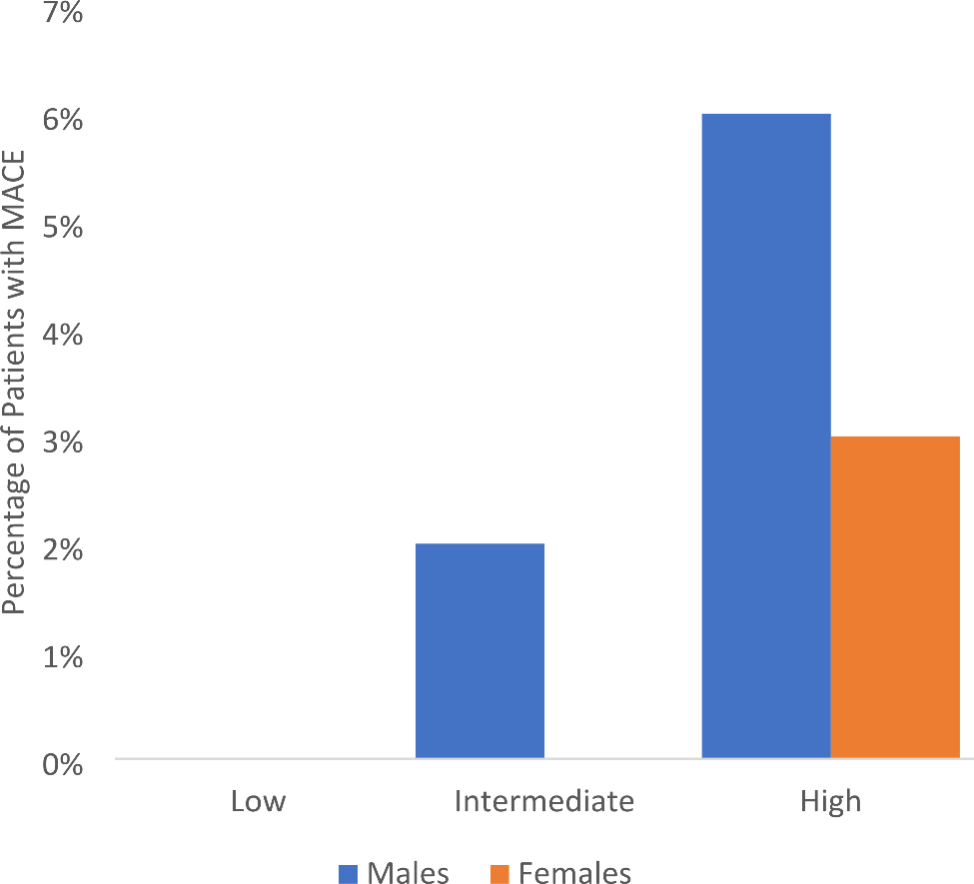
Percentage of males and females with 30-day MACE stratified by HEART score risk category

Low risk patients underwent comparatively fewer non-invasive and invasive studies than intermediate or high-risk patients (Table 2). High-risk patients had the highest order rate of both non-invasive and invasive studies, when compared to low or intermediate risk patients. No differences were seen in non-invasive order rates between males and females across all risk categories. High-risk males underwent more coronary angiogram (33% vs. 16%, *p* = 0.01) and PCI (18% vs. 8%, *p* = 0.04) than high-risk females. There were no differences in coronary angiogram/PCI for males or females categorized as low or intermediate risk. Males experienced more 30-day MACE than females (8% vs. 3%, *p* = 0.001), which was driven primarily by the high-risk category (6% of high-risk males vs. 3% of high-risk females, *p* = 0.02, Fig. 3).

**Table 2:** Primary Outcomes Stratified by Patient Sex and Risk

Outcomes	Males		Females		*p*-value
**Total Low Risk, No. (%)**	N = 101 (18%)		N = 85 (17%)		
CTA cardiac	3	(3%)	2	(2%)	> 0.99
Stress Testing	0	(0%)	2	(2%)	> 0.99
Coronary Angiogram	2	(2%)	0	(0%)	> 0.99
PCI	1	(1%)	0	(0%)	> 0.99
CABG	0	(0%)	0	(0%)	> 0.99
**Total Intermediate Risk, No. (%)**	N = 362 (64%)		N = 340 (67%)		
CTA cardiac	11	(3%)	9	(3%)	0.75
Stress Testing	22	(6%)	22	(6%)	0.83
Coronary Angiogram	19	(5%)	14	(4%)	0.48
PCI	5	(1%)	1	(0.3%)	0.22
CABG	1	(0.3%)	2	(1%)	0.61
**Total High Risk, No. (%)**	N = 104 (18%)		N = 86 (17%)		
CTA cardiac	6	(6%)	3	(3%)	0.52
Stress Testing	18	(17%)	11	(13%)	0.39
Coronary Angiogram	34	(33%)	14	(16%)	0.01
PCI	19	(18%)	7	(8%)	0.04
CABG	5	(5%)	1	(1%)	0.22

CTA cardiac = cardiac computed tomography angiography;

Stress Testing = stress echocardiogram or nuclear perfusion study;

PCI = percutaneous coronary intervention; CABG = coronary artery bypass grafting

## Discussion

This study retrospectively evaluated a university-based CDP to determine sex-based differences in risk stratification, non-invasive or invasive workups, and 30-day MACE. We found no sex-based differences when HEART score was used for risk stratification. Moreover, we found similar rates of non-invasive testing across all risk categories, even when stratified by sex. However, high-risk males underwent comparatively higher rates of coronary angiogram and were found to have higher overall MACE than females at 30-days.

Our study is unique in that we found no differences in risk stratification between males and females, but also in the breakdown of low, intermediate, and high-risk patients. This contradicts other studies that have found that females are inappropriately stratified into lower risk categories [2, 7]. Compared to these studies, we risk-stratified patients as more high-risk. This may have been a ramification of our institutions high-sensitivity assays and sex-specific troponin thresholds, which may have improved sensitivity compared to the contemporary assays used in the other trials [4, 8].

We also identified no differences in non-invasive testing between males and females of all risk categories. This contradicts other studies that have demonstrated disparate testing rates [1, 9]. Interestingly, our study showed very low testing rates for low-risk patients when the CDP was used. This illustrates the utility in CDPs for promoting early discharge and minimizing unnecessary testing. Moreover, a structured and selective approach to testing may reduce downstream interventions without increased acute myocardial infarction [10]. There were otherwise similar ordering patterns for coronary CTAs amongst all risk-categories, but more stress testing in the high-risk category. Current sub-testing recommendations suggest use of coronary CTA in appropriate patients, but exclusion criteria were often met in this population, including previous PCI or CABG, reduced renal function, elevated BMI, irregular heart rhythm, and local preference for functional testing. While there is some data to suggest coronary CTAs may hasten early discharge [11], there is other data to suggest that coronary CTAs lead to more invasive cardiac procedures compared to stress testing [12] without any changes in all-cause mortality. More research is needed to see how these tests differ based on underlying risk categorization.

Review of invasive testing and intervention rates indicate high-risk males undergo comparatively more procedures. There were two instances of coronary angiogram in low-risk males in our review, one of which resulted in PCI. The first patient developed chest pain with syncope and ST-elevations thought to be due to early repolarization. This patient did not develop any troponin elevations greater than the URL, nor did they have any angiographically significant stenoses on coronary angiogram. The second patient had past medical history of STEMI treated with PCI to the LAD. He represented two years later for syncope with brief chest pressure. ECG was normal on admission. He was taken to the catheterization lab due to up-trending troponins where he was found to have non-occlusive thrombus adjacent to his proximal LAD stent. He required repeat stent placement to the proximal LAD. Excluding these two patients, the connection between high-risk males and increased rate of invasive procedures/interventions may be explained by the underlying physiology leading to patient presentation. One study showed that males had more non-culprit lesions by angiography and IVUS, as well as a higher incidence of plaque rupture (16.3% vs. 6.6%, *p* = 0.002) [13]. This was thought to be due in part to plaque structure, where females had less necrotic cores. On the other hand, females demonstrated high rates of microvascular dysfunction and spontaneous coronary artery dissection, which are not necessarily as amenable to PCI [1]. It should be noted that the HEART score is meant to risk stratify patients for ACS, which is not necessarily the same metric as stratifying patients who would benefit from invasive imaging or interventions. This could explain the discordance between invasive testing/interventions rates in high-risk patients.

In conclusion, our retrospective analysis showed no differences in risk stratification or non-invasive testing between males and females when the CDP was used. High risk males, however, underwent more invasive testing and consequently experienced more MACE than females. We also found a higher rate of PCI in high-risk males than females, likely due to sex-based differences in the pathophysiology driving each patient’s presentation. Future work will examine different iterations of the CDP to elucidate how these sex-disparities are being mitigated.

## Study limitations

This study has several limitations. Firstly, it was conducted at a single, academic center with access to testing modalities that may not be available at other institutions. Furthermore, our institution utilized a “cardiac sub-testing pathway,” which recommends coronary CTA in appropriate patients. Exclusion criteria, such as previous PCI or CABG, reduced renal function, elevated BMI, or irregular heart rhythm, were often met. Test selection therefore depended upon available resources, practitioner preference, and shared decision making with the patient, limiting the external validity of our study. Similarly, our institution does not offer stress MRI or PET, which may be the standard modality at other institutions. Secondly, HEART scores were calculated retrospectively by a single reviewer with access to all clinical information. Results may be influenced by the subjective nature of the single reviewer. Moreover, practitioners do not have the same information available at the time of score calculation, resulting in possible incorporation bias. Thirdly, despite having a larger portion of high-risk patients, there were fewer 30-day MACE events than reported in comparable studies. This may be a function of the broad inclusion of all suspected ACS cases, while further restriction based on adjudication would alter these results.

### Electronic supplementary material

Below is the link to the electronic supplementary material.

Supplementary Material 1

## Data Availability

All data generated or analyzed during this study are included in this published article.
